# Clinical research on RSV prevention in children and pregnant women: progress and perspectives

**DOI:** 10.3389/fimmu.2023.1329426

**Published:** 2024-01-24

**Authors:** Xuejia Gong, Erdan Luo, Li Fan, Wanggang Zhang, Yan Yang, Yuhua Du, Xiao Yang, Shasha Xing

**Affiliations:** ^1^ Good Clinical Practice Department, Chengdu Women’s and Children’s Central Hospital, School of Medicine, University of Electronic Science and Technology of China, Chengdu, China; ^2^ Medical Department, Chengdu Women’s and Children’s Central Hospital, School of Medicine, University of Electronic Science and Technology of China, Chengdu, China; ^3^ Obstetrical Department, Chengdu Women’s and Children’s Central Hospital, School of Medicine, University of Electronic Science and Technology of China, Chengdu, China

**Keywords:** RSV vaccines, monoclonal antibody, clinical trial, children, pregnant women

## Abstract

Respiratory syncytial virus (RSV) is a significant causative agent of bronchitis and pneumonia in infants and children. The identification and structural analysis of the surface fusion glycoprotein of RSV represents a pivotal advancement in the development of RSV prevention. This review provides a comprehensive summary of RSV monoclonal antibody (mAb) and vaccine clinical trials registered on ClinicalTrials.gov, emphasizing on the classification, name, target, phase, clinical outcomes, and safety data of RSV vaccination in newborns, infants and children. We also discuss the characteristics of the types of RSV vaccines for maternal immunity and summarize the current clinical research progress of RSV vaccination in pregnant women and their protective efficacy in infants. This review will provide new ideas for the development of RSV prevention for children in the future.

## Introduction

Respiratory syncytial virus (RSV) is the primary causative agent of bronchitis and pneumonia in infants (1), and is also the most common cause of severe lower respiratory tract infections (LRTIs) in infants under 6 months of age, with almost all children being infected by the age of 2 years and reinfection being commonplace (2). According to a report from 2005, the number of fatalities resulting from RSV infection or related complications was estimated to range between 66,000 and 160,000 among children under the age of 5 years (3). Despite the high incidence of RSV, there is currently no definitive treatment for the virus.

**Figure 1:**
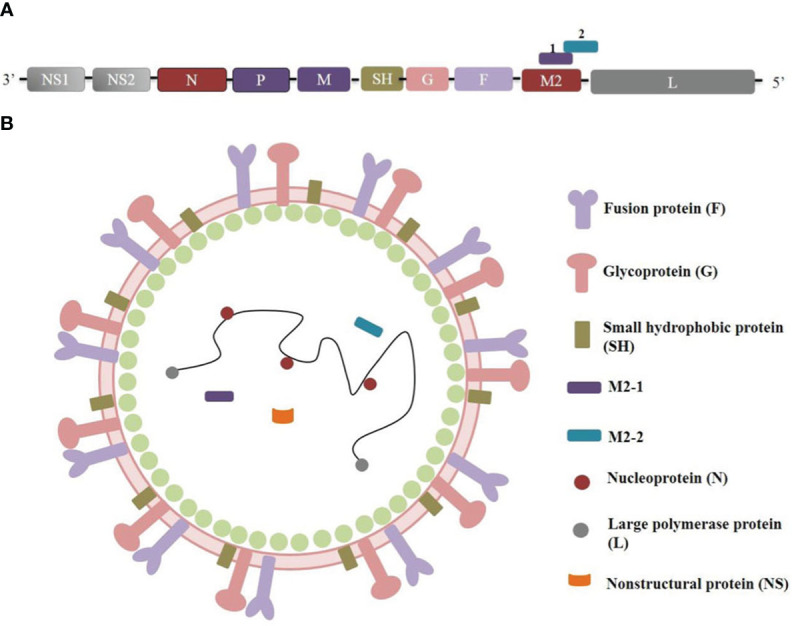
RSV genome organization and protein structure. **(A)** RSV genome orgnizaiton. **(B)** RSV protein structure.

The RSV genome, which spans 15.2 kb (as depicted in **Figure 1**), comprises 10 genes that encode 11 proteins. Notably, the M2 gene contains two overlapping ORFs, resulting in the generation of both M2-1 and M2-2 proteins (4). While M2-1 is essential for virus transcription (5), and M2-2 governs the transition from transcription to genome replication (6). The initial transcription of the RSV genome yields the nonstructural proteins NS1 and NS2, which act in concert to inhibit apoptosis and interferon responses (7, 8). Additionally, the RSV virion is characterized by a single-stranded, negative-sense RNA genome that is enveloped by a lipid bilayer displaying fusion (F), attachment (G), and small hydrophobic (SH) proteins (**Figure 1**). The virion surface is predominantly composed of G and F glycoproteins, which play crucial roles in the entry process (9). The prefusion conformation of F protein is the primary target of RSV-neutralizing activity in human sera, with numerous potent antibodies exhibiting specificity toward this conformation (10). Consequently, structure-based engineering strategies have been prioritized in the development of F protein-containing vaccine candidates to stabilize the F protein in their prefusion conformation (11, 12).

Recent years have witnessed notable advancements in comprehending the configuration and operation of RSV glycoproteins, as well as their interplay with host cytokines that facilitate host cell entry, thereby presenting a new avenue for impeding RSV (13). To date, strategies for RSV preventive interventions encompassed mAbs and six types of vaccines, including particle-based vaccines, vector-based vaccines, live-attenuated or chimeric vaccines, subunit vaccines, mRNA vaccines (14).

This article presents a comprehensive overview of the current clinical research advancements in RSV vaccines and mAbs for newborns, infants, and children. Considering the importance of maternal-fetal immunity, we also include clinical research on RSV vaccines for pregnant women. The discussion encompasses the mechanisms of action of various vaccines, their scope of application, effectiveness, and possible adverse reactions, drawing on data from Clinical Trials.gov and PubMed. The review aims to provide noteworthy insights and innovative perspectives to government policy-makers, academic establishments, and pharmaceutical enterprises.

## Materials and methods

**Figure 2:**
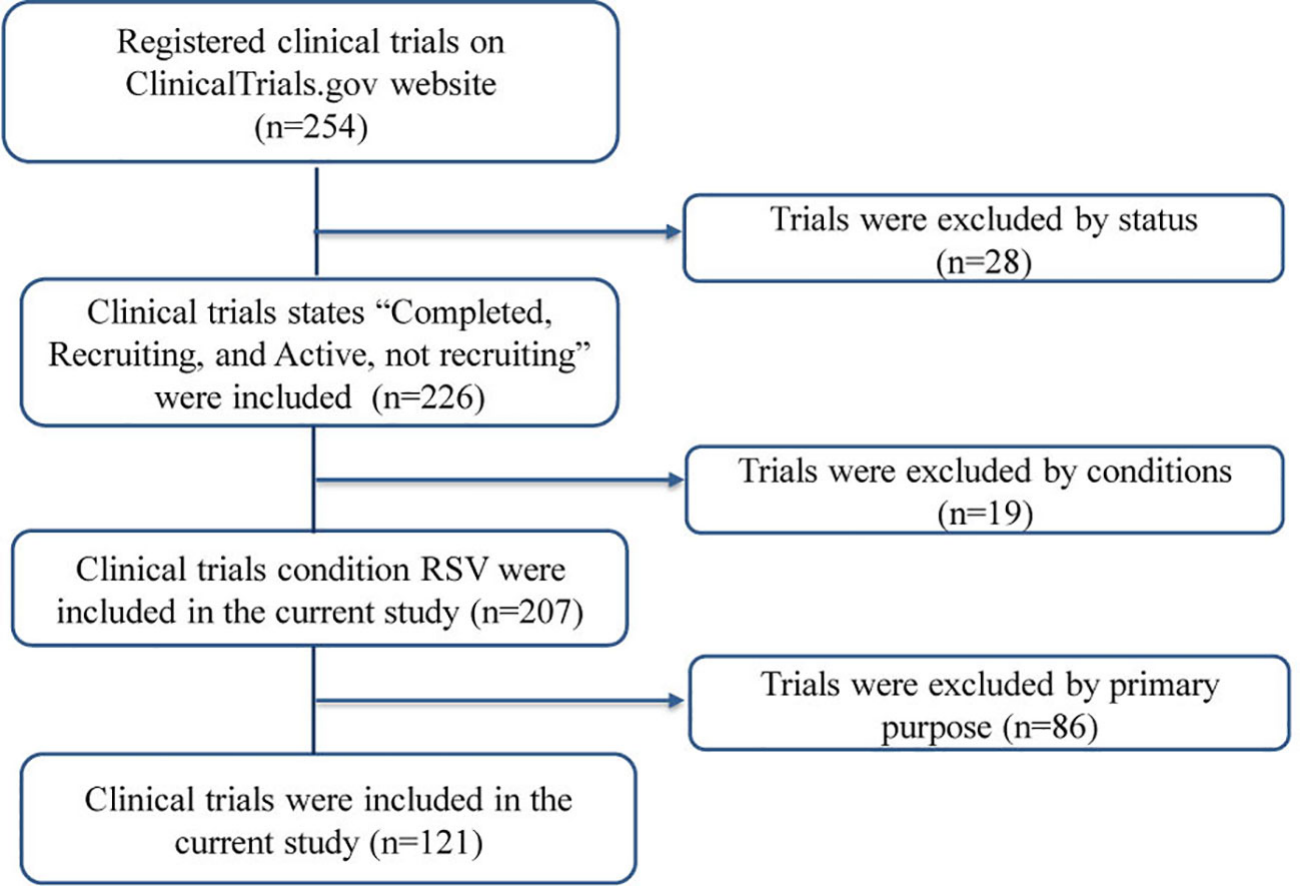
The process of data retrieval.

The ClinicalTrials.gov website was searched using the keywords “respiratory syncytial virus” AND “Early Phase 1, Phase 1, Phase 2, Phase 3, and Phase 4” in the “Additional Criteria” field, with the study start time limited to prior to 20 May 2025. The trial exclusion criteria were as follows: (1) the status was suspended, terminated, withdrawn, or unknown, and (2) the studied condition was not RSV, and (3) the primary purpose was treatment, basic science, or other. A total of 254 clinical trials were screened, with 28 trials excluded based on their status, 19 trials excluded based on their studied condition, and 86 trials excluded based on their primary purpose. Ultimately, 121 clinical trials were included in the analysis. The data-retrieval process is depicted in **Figure 2**.

The following information was collected for analysis: NCT number, intervention, drug, classification, administration, status, sponsor/collaborator, sex, age, phase, funding, date first posted, and location. Descriptive analyses employed numerical and percentage representations for categorical variables, while data processing and analysis were conducted using Microsoft Office Excel 365. As the study solely utilized publicly available data without personal information, it was exempt from ethics committee review.

## Results

### Basic characteristics

A total of 121 trials were included, whose basic characteristics are described in **Table 1**. Most of the RSV vaccine trials were completed, accounting for 67.77%, while 14.05% of trials were in the recruiting, and 17.36% were active but not in the recruiting. The details of these trials are shown in **Table 2**.

**Table 1:** Basic characteristics of trials (n=121).

Characteristics	No. (%)
Vaccine type	
Subunit Vaccine	47 (38.84)
Live-Attenuated Vaccine	26 (21.49)
Vector-based Vaccine	23 (19.01)
MAbs Vaccine	19 (15.70)
mRNA Vaccine	5 (4.13)
Particle-based Vaccine	1 (0.83)
Recruitment status	
Completed	82 (67.77)
Active, not recruiting	21 (17.36)
Recruiting	17 (14.05)
Not yet recruiting	1 (0.83)
Gender of subjects	
All	103 (85.12)
Female	16 (13.22)
Male	2 (1.65)
Age of subjects	
Adult	74 (61.16)
Child	42 (34.71)
Child and Adult	5 (4.13)
Enrollment of subjects	
≤50	29 (23.97)
51-100	22 (18.18)
101-500	24 (19.83)
501-1000	15 (12.40)
1001-10000	22 (18.18)
>10000	9 (7.44)
Funding source	
Industry	99 (81.82)
NIH	16 (13.22)
Other	6 (4.96)
No. of site	
1	32 (26.45)
≥2	88 (72.73)
Not reported	1 (0.83)
Phase	
Phase 1	49 (40.50)
Phase 1|Phase 2	11 (9.09)
Phase 2	25 (20.66)
Phase 2|Phase 3	4 (3.31)
Phase 3	32 (26.45)
Intervention model	
Parallel Assignment	105 (86.78)
Sequential Assignment	11 (9.09)
Single Group Assignment	4 (3.31)
Crossover Assignment	1 (0.83)
Allocation	
Randomized	115 (95.04)
Non-randomized	4 (3.31)
Not reported	2 (1.65)
Masking	
Open label	17 (14.05)
Single Blind	3 (2.48)
Double	15 (12.40)
Triple	29 (23.97)
Quadruple	56 (46.28)
Not reported	1 (0.83)

**Table 2:** The overview of RSV vaccine in children and pregnancy women.

NCT Number	Drugs	Administration	Dose	Age	Phases	First Posted	Reference
NCT01006629	Palivizumab	Intramuscular	15 mg/kg	up to 2 Years	Phase 2/3	3-Nov-09	NA
NCT01466062	Palivizumab	Intramuscular	15 mg/kg	up to 24 Months	Phase 3	6-Nov-11	NA
NCT00192465	Motavizumab	Intravenous;intramuscular	3 mg/kg; 15 mg/kg; 30 mg/kg	18 Years to 49 Years	Phase 1	19-Sep-05	NA
NCT00113490	Motavizumab	Intramuscular	15 mg/kg	up to 24 Months	Phase 1/2	9-Jun-05	NA
NCT00121108	Motavizumab	Intramuscular	15 mg/kg	0 Months to 6 Months	Phase 3	21-Jul-05	(15)
NCT00129766	Motavizumab	Intramuscular	15 mg/kg	up to 24 Months	Phase 3	12-Aug-05	(15)
NCT00316264	Motavizumab	Intramuscular	15 mg/kg	up to 24 Months	Phase 2	20-Apr-06	(16)
NCT02114268	Nirsevimab	Intravenous/Intramuscularly	Intravenously (300, 1,000, or 3,000 mg) Intramuscularly (100 or 300 mg)	18 Years to 49 Years	Phase 1	15-Apr-14	(17)
NCT04484935	Nirsevimab	Intramuscular	50 mg/100 mg	0 Years to 2 Years	Phase 2	24-Jul-20	NA
NCT02878330	Nirsevimab	Intramuscular	50 mg	up to 365 Days	Phase 2	25-Aug-16	(18)
NCT03959488	Nirsevimab	Intramuscular	50 mg or 100 mg	0 Years to 1 Year	Phase 2/3	22-May-19	(19)
NCT05110261	Nirsevimab	Intramuscular	50 mg or 100 mg	0 Years to 1 Year	Phase 3	5-Nov-21	NA
NCT05437510	Nirsevimab	Intramuscular	50 mg or 100 mg	0 Days to 12 Months	Phase 3	29-Jun-22	NA
NCT03979313	Nirsevimab	Intramuscular	50 mg or 100 mg	0 Years to 1 Year	Phase 3	7-Jun-19	(20)
NCT03524118	Clesrovimab	Intramuscular	20 mg; 50 mg; 75 mg	2 Weeks to 8 Months	Phase 1/2	14-May-18	NA
NCT04086472	Clesrovimab	Intravenous	100 mg; 200 mg; 300 mg; 900 mg	18 Years to 55 Years	Phase 2	11-Sep-19	(8)
NCT04767373	Clesrovimab	Intramuscular	100 mg	up to 1 Year	Phase 2/3	23-Feb-21	NA
NCT04938830	Clesrovimab	Intramuscular	100 mg	up to 1 Year	Phase 3	24-Jun-21	NA
NCT05630573	TNM001	Intramuscular	100 mg	up to 1 Year	Phase 1/2	29-Nov-22	NA
NCT01893554	RSV ΔNS2 Δ1313 I1314L Vaccine	Intranasal	0.25 mL per nostril for a total of 0.5 mL	4 Months to 59 Months	Phase 1	9-Jul-13	(21)
NCT03227029	RSV ΔNS2 Δ1313 I1314L Vaccine	Intranasal	10^6 PFU	6 Months to 24 Months	Phase 1	24-Jul-17	(22)
NCT03916185	RSV ΔNS2 Δ1313 I1314L Vaccine	Intranasal	10^6 PFU	6 Months to 24 Months	Phase 1/2	16-Apr-19	NA
NCT04227210	MV-012-968	Intranasal	–	18 Years to 40 Years	Phase 1	13-Jan-20	NA
NCT046903351	MV-012-968	Intranasal	1 x10^6 PFU	18 Years to 45 Years	Phase 2	30-Dec-20	NA
NCT04444284	MV-012-968	Intranasal	–	15 Months to 59 Months	Phase 1	23-Jun-20	NA
NCT04909021	MV-012-968	Intranasal	–	6 Months to 36 Months	Phase 1	1-Jun-21	NA
NCT02237209	RSV LID ΔM2-2	Intranasal	0.5 mL	6 Months to 24 Months	Phase 1	11-Sep-14	(23)
NCT02040831	RSV LID ΔM2-2	Intranasal	0.5 mL	6 Months to 24 Months	Phase 1	20-Jan-14	(11)
NCT01459198	RSV MEDI ΔM2-2 vaccine	Intranasal	0.5 mL	6 Months to 49 Years	Phase 1	25-Oct-11	(12)
NCT04520659	RSV LID ΔM2-2 1030s	Intranasal	0.5 mL	6 Months to 24 Months	Phase 1	20-Aug-20	NA
NCT02794870	RSV LID ΔM2-2 1030s	Intranasal	0.5 mL	6 Months to 24 Months	Phase 1	9-Jun-16	NA
NCT02952339	RSV LID ΔM2-2 1030s	Intranasal	0.5 mL	6 Months to 24 Months	Phase 1	2-Nov-16	NA
NCT02601612	D46cpΔM2-2	Intranasal	0.5 mL	6 Months to 60 Months	Phase 1	26- Nov-15	NA
NCT03102034	D46/NS2/N/ΔM2-2-HindIII	Intranasal	0.5mL	6 Months to 24 Months	Phase 1	5-Apr-17	(24)
NCT03099291	D46/NS2/N/ΔM2-2-HindIII	Intranasal	0.5mL	6 Months to 24 Months	Phase 1	4-Apr-17	(24)
NCT01852266	RSV cps2 Vaccine	Intranasal	0.5 mL	6 Months to 24 Months	Phase 1	13-May-13	(25)
NCT01968083	RSV cps2 Vaccine	Intranasal	0.5 mL	6 Months to 24 Months	Phase 1	23-Oct-13	(25)
NCT04295070	CodaVax-RSV	Intranasal	10^4 PFU; 10^5 10^6 PFU	50 Years to 75 Years	Phase 1	4-Mar-20	NA
NCT04919109	CodaVax-RSV	Intranasal	10^4 PFU; 10^5 10^6 PFU	6 Months to 5 Years	Phase 1	9-Jun-21	NA
NCT00493285	MEDI-534	Intranasal	–	6 Months to 23 Months	Phase 1	28-Jun-07	NA
NCT05687279	RSVt Vaccine	Intranasal	–	6 Months to 23 Months	Phase 1/2	18-Jan-23	NA
NCT04491877	VAD00001	Intranasal	–	6 Months to 18 Months	Phase 2	29-Jul-20	NA
NCT03596801	6120/ΔNS1	Intranasal	0.5mL	6 Months to 59 Months	Phase 1	24-Jul-18	NA
NCT03387137	RSV 6120/ΔNS2/1030s	Intranasal	0.5mL	6 Months to 60 Months	Phase 1	29-Dec-17	NA
NCT04138056	RSVPreF3 (RSV MAT 009)	Intramuscular	300 mg; 500 mg; 120mg	18 Years to 45 Years	Phase 2	24-Oct-19	NA
NCT04605159	RSVPreF3 (RSV MAT 009)	Intramuscular	–	18 Years to 49 Years	Phase 3	27-Oct-20	NA
NCT04126213	RSVPreF3	Intramuscular	60 μg; 120 μg	18 Years to 40 Years	Phase 2	15-Oct-19	(26)
NCT04980391	RSVPreF3 (RSV MAT 009)	Intramuscular	–	15 Years to 49 Years	Phase 3	28-Jul-21	NA
NCT05169905	RSVPreF3 (RSV MAT 009)	Intramuscular	–	9 Years to 49 Years	Phase 3	27-Dec-21	NA
NCT05045144	GSK3888550A	Intramuscular	120μg	18 Years to 49 Years	Phase 3	16-Sep-21	NA
NCT03674177	GSK3888550A	Intramuscular	30 μg; 60 μg; 120 μg	18 Years to 45 Years	Phase 1	17-Sep-18	(27)
NCT04032093	RSVPreF	Intramuscular	0.5 mL	18 Years to 49 Years	Phase 2	25-Jul-19	(28)
NCT04071158	RSVPreF	Intramuscular	0.5 mL	18 Years to 49 Years	Phase 2	28-Aug-19	(29)
NCT04424316	RSVPreF	Intramuscular	120 μg	0 Years to 49 Years	Phase 3	9-Jun-20	(30)
NCT02956837	GSK3003891A	Intramuscular	30 μg; 60 μg; 120 μg	18 Years to 45 Years	Phase 2	6-Nov-16	(31)
NCT02753413	GSK3003891A	Intramuscular	30 µg; 60 µg	18 Years to 45 Years	Phase 2	27-Apr-16	(32)
NCT02360475	GSK3003895A	Intramuscular	30 µg; 60 µg	18 Years to 45 Years	Phase 2	10-Feb-15	(32)
NCT02624947	RSV F Vaccine	Intramuscular	0.5mL	18 Years to 40 Years	Phase 3	9-Dec-15	(33)
NCT02247726	RSV F Vaccine	Intramuscular	0.5mL	18 Years to 40 Years	Phase 2	25-Sep-14	(34)
NCT02296463	RSV F Vaccine	Intramuscular	0.5mL	24 Months to 72 Months	Phase 1	20-Nov-14	NA
NCT02491463	GSK3389245A/ChAd155-RSV	Intramuscular	–	18 Years to 45 Years	Phase 1	8-Jul-15	(35)
NCT03636906	GSK3389245A	Intramuscular	–	6 Months to 7 Months	Phase 1|Phase 2	17-Aug-18	NA
NCT02927873	GSK3389245A	Intramuscular	0.5ml; 0.15mL	12 Months to 23 Months	Phase 1/2	7-Oct-16	NA
NCT03606512	Ad26.RSV.preF	Intramuscular	2.5*10^10 vp	12 Months to 24 Months	Phase 1/2	31-Jul-18	NA
NCT05655182	BLB-201	Intranasal	10^6 PFU; 10^7 PFU	6 Months to 5 Years	Phase 1|Phase 2	19-Dec-22	NA
NCT04528719	mRNA-1345	Intramuscular	–	12 Months to 79 Years	Phase 1	27-Aug-20	NA
NCT04519073	V-306 candidate vaccine	Intramuscular	15 µg; 50 µg; 150 µg	18 Years to 45 Years	Phase 1	19-Aug-20	NA

The types of RSV vaccines examined included 47 subunit vaccines (38.84%), 26 live-attenuated vaccines (21.49%), 23 vector-based vaccines (19.01%), 19 mAbs vaccines (15.70%), 5 mRNA vaccines (4.13%) and 1 particle-based vaccine (0.83%).

**Figure 3:**
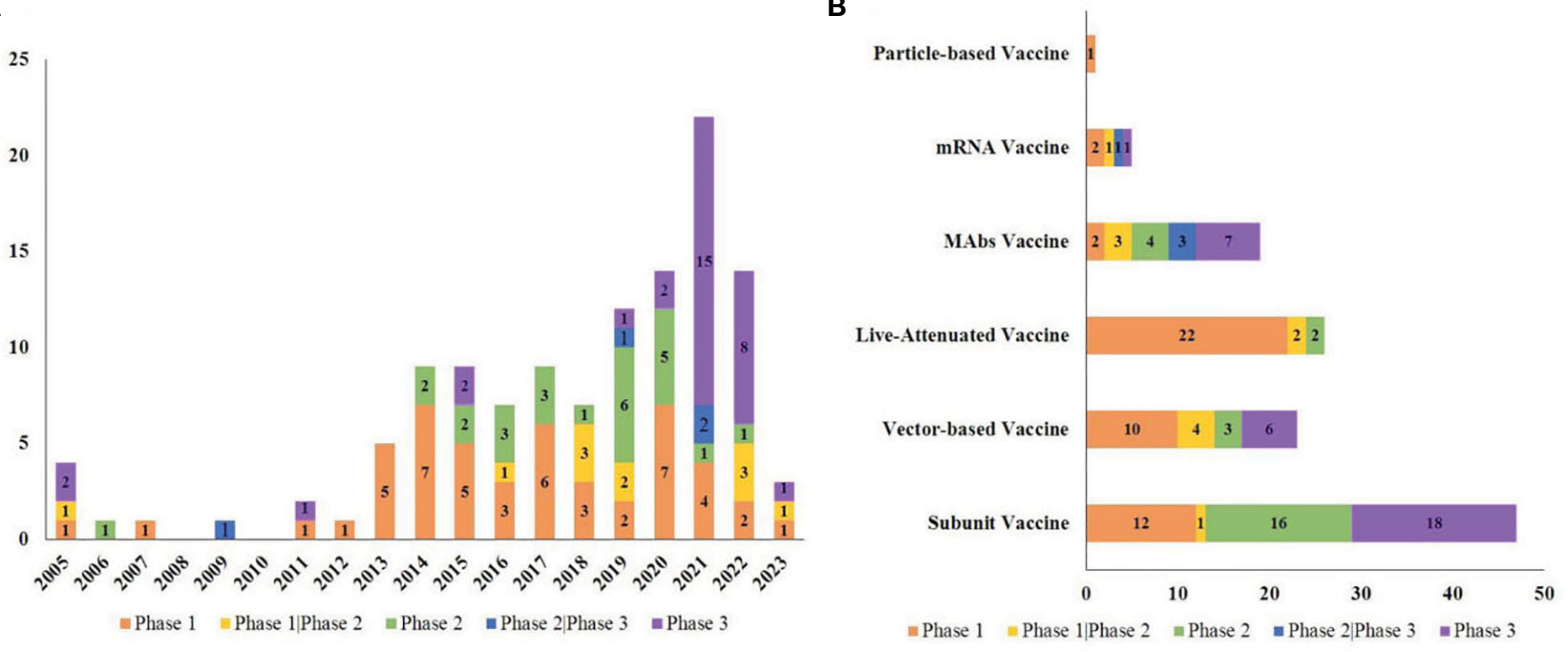
Overview of RSV vaccine trials. **(A)** The number of trials in each phase per year from 2005 to 2023; **(B)** The number of trials in each phase in various vaccine types.

Most of the trials (85.12%) were conducted among both sexes, and 13.22% were conducted only among females. There were 42 trials conducted only among children (34.71%), 74 trials conducted among adults (61.16%), and 5 trials conducted among both children and adults (4.13%). An overview of RSV clinical research is shown in **Figure 3**.

**Figure 4:**
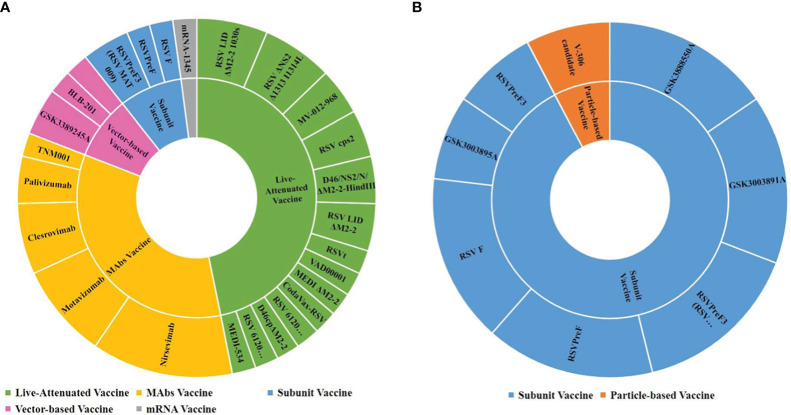
RSV vaccine types and drugs in trials among children and pregnant women. **(A)** Children; **(B)** Pregnant women.

From the aspect of the subject population, 47 trials focused on children and 13 focused on pregnant women. It was found that the RSV vaccines used in pregnant women were subunit vaccines and particle-based vaccines, while those used in children included subunit vaccines, live-attenuated vaccines, vector-based vaccines and mRNA vaccines and mAbs. **Figures 4A, B** shows the drug details for each kind of vaccine and mAbs.

### RSV monoclonal antibody for children

#### mAbs

mAbs present a compelling alternative for addressing viral infections, given their remarkable specificity toward pathogens and ability to provide passive immune responses (36). The primary objective is to target a highly neutralizing, sensitive epitope situated on the RSV pre-F protein (37–39). Furthermore, the latest generation of RSV antibodies undergoes Fc mutation modification, which can prolong their half-life. There are 5 mAbs with activity against RSV, including palivizumab, motavizumab, nirsevimab, clesrovimab, and TM001.

(1) Palivizumab. Palivizumab (Synagis) is the first humanized mAb approved by the FDA for the prophylaxis of high-risk of hospital admission infants during bronchiolitis season (40, 41). Palivizumab is administered intramuscularly at 15 mg/kg every month during five months of the first RSV season to prevent serious RSV LRTI in children (42). Palivizumab binds antigenic site A, a highly conserved region on the RSV fusion (F) protein, and can block the membrane fusion process by binding to RSV fusion proteins on the surface of the virus, and can also prevent the fusion process between cells infected with RSV (43). The intervention is restricted to a narrow subset of the pediatric population, infants with a gestational age below 35 weeks and infants up to 6 months of age at the time of RSV season onset. This leaves millions of infants at risk of severe or potentially fatal disease every year, without protection from RSV. Two clinical trials are currently underway to investigate the preventive efficacy of palivizumab against RSV in children under two years of age (NCT01006629/phase 2/3; NCT01466062/phase 3). In light of the advent of novel long-acting mAbs, it is pertinent to deliberate upon the necessity of conducting additional experiments to verify the protective effect on children over 6 month old in this regard.

#### Motavizumab

Motavizumab, which is alternatively referred to as MEDI-524 or Numax, is a second-generation mAb that is developed from palivizumab through affinity maturation techniques (44). It has been found to effectively reduce the disease burden of RSV in high-risk infants (35). This mAb has been the subject of five clinical trials. A randomized, double-blind, placebo-controlled trial (NCT00121108/phase 3) (15) indicated that motavizumab led to a relative reduction of 87% in the proportion of infants hospitalized due to RSV when compared to the placebo group. Moreover, the incidence of adverse events (AEs) was lower among subjects administered motavizumab than among those who received placebo, and no fatalities associated with motavizumab were reported. There was no statistically significant difference observed in the occurrence of severe adverse events (SAEs) or hypersensitivity events between the two groups. When comparing the administration of motavizumab alone to a combined regimen of motavizumab and palivizumab, it was observed that the rates of AEs and the antidrug antibodies (ADAs) associated with the sequential administration of the combined regimen to high-risk children were similar between the two groups (16).

#### Nirsevimab

Nirsevimab, a human mAb designed to target site Ø of the F protein and that features a YTE mutation in the Fc portion to prolong its serum half-life, when administered as a singular dosage, offers comprehensive protection throughout a RSV season (45). In November 2022, the EMA and the EU granted approval for the use of Beyfortus (nirsevimab) in newborns and infants to aid in the prevention of RSV LRTIs during their initial RSV pandemic season.

The mean half-life of MEDI8897 ranged from 85 to 117 days among different dosage cohorts, accompanied by a bioavailability of 77% following subsequent to the administration of a 300-mg i.m. dose (NCT02114268/phase1) (17). The incidences of ADA and AEs in the intervention group were comparable to those observed in the placebo group. Subsequent phase 2 (NCT02878330) (18) and phase 3 trials (NCT03979313) (20) demonstrated that nirsevimab exhibited efficacies of 70.1% (95% CI, 52.3 to 81.2) and 74.5% (95% CI, 49.6 to 87.1) in treating LRTIs in drug-treated patients, respectively. Additionally, the efficacy of nirsevimab in preventing hospitalization due to RSV in premature infants was found to be 78.4% (95% CI, 51.9 to 90.3) and 62.1% (95% CI, -8.6 to 86.8), respectively. SAEs were similar between the two groups. In addition, the safety profiles of nirsevimab in infants diagnosed with congenital heart disease (CHD) or chronic lung disease (CLD) were comparable to those observed in preterm infants receiving palivizumab(NCT03959488/phase 2/3) (19).

#### Clesrovimab (MK-1654)

Clesrovimab is a mAb with an extended half-life and the same YTE mutation as that in nirsevimab and is designed to target site IV of the RSV F protein, although it partially targets site V and preferentially binds to pre-F. MK-1654 was tested in two phase 1 clinical trials conducted among Japanese adults. A single intramuscular injection of MK-1654 produced a dose-dependent increase in RSV serum-neutralizing antibody (SNA) titers (46). The antibody exhibited a half-life ranging from 73 to 88 days, with an estimated bioavailability of 69% at the 300-mg intramuscular dose. There was no significant difference in safety events between the MK1654 and placebo groups. Orito et al. (47) proved that the bioavailability of MK-1654 in healthy adults was 86% and 77% for the 100 mg i.m. and 300 mg i.m. doses, respectively. The registration numbers were not found in clinicaltrials.gov. Currently, this mAb is being tested in 7 trials in infants.

### RSV vaccines for children

#### LAVs

LAVs are promising options for young children due to their intranasal administration, ability to induce both local mucosal and systemic responses, and immunogenicity when maternal antibodies are present, thereby enabling the immunization of vulnerable infants at high risk of severe illness (48). Enhanced comprehension of the RSV genome and reverse genetics has facilitated the logical development of LAV candidates through the elimination or alteration of proteins that are recognized to exert a noteworthy influence on the control of RNA biosynthesis or hinder the host immune reactions, such as NS2, M2-2, SH, L, and G proteins, which resulting in limited viral replication (49, 50). LAVs offer a valuable needle-free option for the active intranasal immunization of older infants who may not receive adequate protection from maternal vaccines or monoclonal antibodies.

#### RSV ΔNS2/Δ1313/I1314L

RSV ΔNS2/Δ1313/I1314L was developed by deleting the NS2 gene and the amino acid S1313 from the L polymerase protein. Consequently, a phenotypically stable variant was obtained, which displays temperature sensitivity under physiological conditions (51). In children who were seronegative for RSV, administration of a 106 plaque forming unit (PFU) dose of RSV/ΔNS2/Δ1313/I1314L was found to be both well tolerated and capable of inducing an immune response. Following the RSV season, it was observed that vaccinated individuals experienced a significant increase in RSV titers (1:955) compared to placebo recipients (1:69), indicating the effectiveness of the vaccine (NCT01893554) (21). Both RSV/ΔNS2/Δ1313/I1314L and RSV/276 vaccine recipients (NCT03227029) (22) experienced upper respiratory illness and/or fever post vaccination, with generally mild symptoms. It is noteworthy that the incidence of cough was significantly higher in subjects that received RSV/276 than in those that received RSV/ΔNS2/Δ1313/I1314L (48% *vs*. 12%). Lower respiratory illnesses and SAEs were not observed. The two groups demonstrated a ≥4-fold elevation in serum RSV neutralizing titers and anti-RSV F IgG titers by 60% and 92%, respectively.

#### MV-012–968

MV-012-968 is a live attenuated vaccine that is free of adjuvants. This vaccine, modifications were made to it’s NS1 and NS2 proteins, as well as the G proteins, with the SH deletion and ablation of secreted G protein. Currently, the safety and immunogenicity of MV-012-968 is being assessed in phase 1 and phase 2 clinical trials, but the results were not published.

#### RSV LID ΔM2-2

RSV LID ΔM2-2 is a candidate vaccine for RSV that involves the deletion of RSV M2-2. The attenuation of LIDΔM2-2 leads to an increase in viral gene transcription and antigen expression, and a decrease in RNA replication. The vaccine virus was found to be shed by 95% of individuals who received the LIDΔM2-2 (105 PFU) vaccine (median peak titers, 3.8 log10 PFU/mL; 6.3 log10 copies/mL)(NCT02237209) (23). Additionally, 90% of vaccine recipients demonstrated a serum-neutralizing antibody (SNA) increase of at least fourfold. It is noteworthy that both the vaccine and placebo groups experienced a high prevalence of respiratory symptoms and fever. Notably, one vaccine recipient exhibited grade 2 rhonchi in conjunction with vaccine shedding, rhinovirus, and enterovirus. Following the RSV season, vaccine recipients (8/19) demonstrated a marked increase in RSV antibody titers compared to placebo recipients (2/9), without experiencing medically attended RSV disease.

#### LID/ΔM2-2/1030s

LID/ΔM2-2/1030s is produced by eliminating of the RSV M2-2 protein and introducing of a genetically stabilized temperature-sensitive mutation, known as 1030s, in the RSV polymerase protein. LID/ΔM2-2/1030s vaccine (105 PFU) (52) exhibited 85% shedding of the vaccine and a ≥4-fold increase in serum-neutralizing antibodies. Respiratory symptoms and fever were commonly reported by vaccine recipients (60%) and placebo recipients (27%). Notably, a vaccinated individual experienced grade 2 wheezing caused by Rhinovirus but did not concurrently shed LID Δ M2-2/1030s.

#### D46/NS2/N/ΔM2-2-HindIII

D46/NS2/N/ΔM2-2-HindIII, a recombinant LAVs with an M2-2 deletion, was derived from the earlier candidate LID/ΔM2-2. All 21 individuals who received D46/NS2/N/ΔM2-2-HindIII (105 PFUs) (NCT03102034, NCT03099291) (24) were found to be infected with the vaccine, with 20 of them (95%) exhibiting vaccine shedding. Notably, 95% and 100% of the vaccine recipients experienced a ≥4-fold increase in serum RSV-neutralizing antibodies and anti-RSV fusion immunoglobulin G, respectively. Additionally, both vaccinees (76%) and placebo recipients (18%) reported mild upper respiratory tract symptoms and/or fever.

#### rA2cp248/404/1030 Δ SH mutation and rA2cp248/104/1030 Delta SH mutation

These two vaccines serve as the initial attenuated candidates for RSV immunization in young children (53). Both vaccines exhibited high attenuation in adults and RSV-seropositive children while also exhibiting favorable tolerability and immunogenicity in RSV-seronegative children. Notably, the replication of rA2cp248/404/1030 Delta SH was found to be restricted in RSV-seronegative children, with a mean peak titer of 10 (2.5) PFUs/mL, compared to 10 (4.3) PFUs/mL for rA2cp248/404 Delta SH. Although rA2cp248/404/1030 Delta SH was well tolerated in infants, only 44% of the recipients who were administered two 10 (5.3) PFU doses of the vaccine exhibited discernible antibody responses.

#### MEDI-559 and RSV cold-passage/stabilized 2

MEDI-559 (54) was mutated from RSV rA2cp248/404/1030 Δ SH and is currently being assessed in phase 1/2 clinical trials (NCT00767416). RSVcps2 is a newly developed vaccine that was modified from MEDI-559 through the incorporation of five nucleotide changes and one amino acid alteration. This modification resulted in the stabilization of two significant decay mutations, thereby preventing their degradation. Administration of RSVcps2 via the intranasal resulted in a significant proportion of vaccinees (85%) acquiring RSVcps2 infection, with 77% shedding the vaccine virus and 59% developing a≥4-fold increase in RSV-SNA titers (NCT01852266, NCT01968083) (25). The incidence of respiratory tract and/or febrile illness was similar between the vaccine and placebo groups.

### Vector-based vaccines

In recombinant vector vaccines, a genetically altered replication-deficient virus is employed to elicit both humoral and cellular immunity through the transmission of genes encoding RSV antigens. Currently, there are 3 vaccine candidates undergoing clinical trials for the pediatric population, namely, ChAd155-RSV derived from chimpanzee adenovectors (GSK3389245A), Ad26.RSV.preF, and PIV5-vectored RSV vaccine (BLB-201).

#### ChAd155-RSV

The ChAd155-RSV vaccine, which is currently under investigation, is a chimpanzee adenovirus-based vaccine that expresses three distinct proteins, namely, fusion proteins, nucleoproteins, and M2-1 proteins (55). Following the administration of the first dose of GSK3389245A (NCT02927873; phase1/2 trial) (56), all groups (0.5 × 1010, 1.5 × 1010, and 5 × 1010 viral particles) exhibited a dose-dependent escalation in RSV-A Nab titers, with no discernible booster effect observed after the second dose. At the one-year mark, RSV-A Nab titers remained elevated above prevaccination levels. The incidence of AEs was comparable across all groups, with the exception of fever, which was more prevalent in the high dose group. The majority of fevers were mild. There were no SAEs and hospitalizations related to vaccine were reported.

#### Ad26.RSV.preF

An adenovirus serotype 26 RSV vector encoding a prefusion F (preF) protein (Ad26.RSV.preF) in combination with an RSV preF protein. Previous studies have demonstrated the effectiveness of Ad26.RSV.preF in mitigating lower respiratory tract disease caused by RSV among the elderly population (57, 58). The geometric mean titers for RSV A2 neutralization in children aged 12-24 months who were administered Ad26.RSV.preF (NCT03606512; phase1/2 trial) (59) exhibited a significant increase from 121 (Day 0) to 1608 (Day 29) and further increased to 2235 (Day 57), persisting at elevated levels for a duration of over 7 months. The administration of Ad26.RSV.preF resulted in a lower incidence of RSV infection in children (1, 4.2%) in comparison to the administration of placebo (5, 41.7%). No SAEs related to vaccination were reported.

#### PIV5-vectored RSV vaccine (BLB-201)

Parainfluenza virus 5 (PIV5) is a paramyxovirus that has been utilized as a vector-based platform for vaccine development. Two distinct recombinant PIV5 viruses have been developed, one expressing the fusion (F) protein and the other expressing the attachment glycoprotein (G). A phase 1 clinical trial is currently underway to evaluate the safety, tolerability, and immunogenicity of the BLB-201 vaccine in children (NCT05655182).

## Others

At present, the safety of mRNA vaccines (mRNA-1345, NCT04528719) and subunit vaccine (NCT02296463) are currently being evaluated in phase 1 clinical trials.

### RSV vaccines in pregnant women

Maternal immunization is regarded as a viable approach to safeguard infants against a diverse range of perilous infections during the initial stages of life through the transfer of maternal immunity (60–62). These vaccine types include subunit-and particle-based vaccines. RSV preF3, RSV preF, and RSV F vaccines are notable examples that have advanced to phase 3 clinical trials and have demonstrated favorable safety, immunogenicity, and maternal-fetal transmission rates, as evidenced by published data. A comprehensive account of these findings is presented in the following sections.

#### RSVPreF3

RSVPreF3 is an adjuvant-free vaccine. There are seven clinical studies on RSVPreF3. In a cohort of healthy nonpregnant females, RSVPreF3(GSK3888550A)(30/60/120 µg) group exhibited a higher incidence of induced local adverse events (AEs) (4%~53.2%) than the placebo group (0% ~ 15.9%). Nonetheless, the majority of the aforementioned events were categorized as mild to moderate. Furthermore, the frequency of unsolicited AE reports was similar between the RSVPreF3 and placebo, and no SAEs associated with the medication were documented (NCT03674177, phase1) (27). Bebia et al. (26) suggest that RSVPreF3 (RSV MAT 009) (60/120 µg) was well tolerated, as no AEs related to the vaccine were observed both in the pregnant women and in their offspring. Furthermore, the level of the maternal nAbs against RSV increased by more than 10 times, even 43 days after labor, with a range of 8.9-10.0 times. The level of nAbs in neonates is at its peak during birth and gradually declines until the 181st day postpartum (NCT04126213/phase2).

#### RSV stabilized prefusion F subunit vaccine

In a cohort of pregnant women were randomly assigned to receive the bivalent RSVpreF vaccines (120/240 μg), with or without aluminum hydroxide, or placebo (NCT04032093/phase2b) (28). The study revealed a significant increase in the titer of 50% nAb in the vaccinated group compared to the placebo group. Furthermore, the transfer ratios of neutralizing antibodies through the placenta varied between 1.41 and 2.10. Furthermore, the incidence of AEs observed in both women and infants in the vaccine group was similar to that in the placebo groups. RSVpreF was well tolerated and safe when administered alone or in conjunction with acellular pertussis vaccine adsorbed (Tdap) in the healthy, nonpregnant woman (NCT04071158/phase2b) (29).

A single intramuscular injection of RSVpreF vaccine or placebo was administered to pregnant women at 24 to 36 weeks gestation (NCT04424316/phase3) (30). Within the first 90 days following birth, the vaccine demonstrated an effective rate of 81.8% (99.5% CI, 40.6 to 96.3) in preventing severe lower respiratory tract diseases caused by medication treatment and a 57.1% (99.5% CI, 14.7 to 79.8) effective rate in preventing lower respiratory tract diseases related to respiratory syncytial virus in the vaccine group. Additionally, within the first 180 days following birth, the effective rate of prophylactic treatment for severe lower respiratory tract diseases was 69.4% (97.58% CI, 44.3 to 84.1). No noticeable safety signals were detected in maternal participants or infants and toddlers up to 24 months of age. The frequency of AEs reported within 1 month following injection or birth was similar between the two groups.

The immunogenicity and safety of GSK3003891A (30/60/120 μg) was assessed in healthy nonpregnant women (NCT02956837/phase2) by a single intramuscular injection (31). The results showed that the geometric mean titer (GMT) and PCA concentration increased in a dose-dependent manner. Another comparative analysis was conducted to evaluate the safety and immunogenicity of the RSV F-020/GSK3003895A (NCT02360475/phase2) and RSV F-024/GSK3003891A vaccines (NCT02753413/phase2) (32). In the clinical trial identified as RSV F-020, participants were randomized and subsequently received a single dose of RSV PreF vaccine (30/60 μg without adjuvant or 60 μg with aluminum adjuvant) or Tdap. In the RSV F-024 trial (NCT02753413/phase2), participants were randomly assigned to receive a single dose of RSV PreF (60 μg without adjuvant) or Tdap. The two studies demonstrated comparable reactogenicity profiles between the RSV-PreF and Tdap vaccines. There have been no SAEs associated with vaccine injection.

#### RSV F vaccine (RSV fusion (F) protein nanoparticle)

The pregnant women who received the RSV F vaccine exhibited a significant elevation in RSV-specific antibody levels, exhibiting responses that were comparable to mAbs known for their specificity towards various RSV-neutralizing epitopes. (NCT02247726/phase2) (34). The transference rate of antibodies through the placenta was discovered to range from 90% to 120% across diverse assays for neonates born to immunized females. Offspring born to mothers who received vaccination exhibited RSV-specific antibodies with half-lives of approximately 40 days, and no instances of severe RSV disease were detected in these neonates.

The pregnant woman was administrated an intramuscular injection of the RSV F vaccine, to assess the efficacy and its potential to provide protection to live-born infants (NCT02624947/phase3) (33). Within the initial 90 days of life, the vaccine effectiveness against RSV-associated, clinically significant LRTIs was 39.4%, and the vaccine efficacy for RSV-associated LRTIs with severe hypoxemia was 48.3%. Moreover, the vaccine’s effectiveness in preventing hospitalization due to RSV-associated LRTIs was determined to be 44.4%. The occurrence of local injection site reactions was observed to be greater in female recipients of the vaccine (40.7% vs 9.9%). Among these mAbs and vaccines, the efficacy and safety data can be retrieved in PubMed are listed in **Table 3**. Based on the attributes associated with the staging of clinical trials, it is reasonable to accord precedence to the dissemination of clinical research findings derived from extensive sample sizes. For instance, Nirsevimab has successfully concluded Phase III clinical trials, thereby furnishing solely Phase III efficacy and safety data.

**Table 3:** The latest results of efficacy and safety details of RSV mAbs and vaccines in children and pregnancy women.

Interventions	Administration	Dose	Phases	Conclusion	AE/SAE	Reference
Motavizumab	Intramuscular	15 mg/kg	phase3	Led to a relative reduction of 87% in the proportion of infants hospitalized due to RSV when compared to the placebo group.	No death deemed to be related to the product; Hypersensitivity events were similar between motavizumab and placebo group; No effect on rates of medically attended wheezing in children aged 1-3 years.	(15)
Nirsevimab*	Intramuscular	300 mg	Phase 3	The incidence of medically attended RSV-associated lower respiratory tract infection was 74.5% lower with nirsevimab prophylaxis than with placebo and the incidence of hospitalization for RSV-associated lower respiratory tract infection was 62.1% lower with nirsevimab than with placebo.	SAE were reported 6.8% in who received nirsevimab and 7.3% in who received placebo.	(17)
RSV ΔNS2 Δ1313 I1314L Vaccine	Intranasal	106 PFUs	Phase 1	Infectious (RSV/ΔNS2/Δ1313/I1314L replication detected in 90% of vaccinees), and immunogenic (geometric mean serum RSV plaque-reduction neutralizing antibody titer, 1:64)	In RSV-seropositive participants, URI was observed in 2 and cough was observed in 1 of 10 vaccinees during the 28-day postimmunization reporting period in each case, rhinovirus was detected in NW samples at the time of illness. None of the vaccinees shed vaccine virus, indicative of attenuation.	(21, 22)
RSV LID ΔM2-2 Vaccine	Intranasal	105 PFUs	Phase 1	Vaccine virus was shed by 95% of vaccinees	Respiratory symptoms and fever were common in vaccine (95%) and placebo (78%)	(23)
D46/NS2/N/ΔM2-2-HindIII	Intranasal	105 PFUs	Phase 1	All 21 vaccinees were infected with vaccine; RSV-MAARI occurred in 2 vaccinees and 4 placebo recipients.	Mild upper respiratory tract symptoms and/or fever occurred in vaccinees (76%) and placebo recipients (18%)	(24)
RSV cps2 Vaccine	All	105.3 PFU	Phase 1	A total of 85% of vaccinees were infected with RSVcps2	The AEs rate of fever and cough occurred similar in vaccine 5 (15) and placebo 1(6)recipients.	(25)
RSVPreF3 vaccine	Intramuscular	60/120 µg	Phase 2	(1) neutralizing antibody (nAb) titers in mothers increased 12.7- and 14.9-fold against RSV-A and 10.6- and 13.2-fold against RSV-B, respectively, 1 month postvaccination and remained 8.9-10.0-fold over prevaccination at day 43 postdelivery; (2) nAb titers were consistently higher compared to placebo recipients; (3) placental transfer ratios for anti-RSVPreF3 antibodies at birth were 1.62 and 1.90, respectively, and (4) nAb levels in infants were highest at birth and declined through day 181 postbirth.	No pregnancy-related or neonatal adverse events of special interest were considered vaccine/placebo related.	(26)
RSVpreF vaccine	Intramuscular	120 μg	Phase 3	Vaccine efficacy: 81.8% within 90 days; 69.4% within 180 days.	The incidences of adverse events reported were similar in the vaccine group and the placebo group.	(30)
RSV F vaccine with adjuvant	Intramuscular	120 μg	Phase 3/phase2	Vaccine efficacy: lower respiratory tract infection, 39.4%; RSV-associated lower respiratory tract infection with severe hypoxemia, 48.3%; hospitalization for RSV-associated lower respiratory tract infection 44.4%.	Local injection-site reactions among the women were more common with vaccine than with placebo (40.7% *vs*. 9.9%), but the percentages of participants who had other adverse events were similar in the two groups.	(33)

National Institute of Allergy and Infectious Diseases (NIAID); GSK3389245A (ChAd155-Vectored RSV Vaccine); PIV5-vectored RSV Vaccine (BLB-201). Plaque-forming units (PFU); RSV fusion (F) protein stabilized in the prefusion conformation (RSVPreF3); RSV prefusion F protein-based (RSVpreF); *was approved by government agency.

## Perspectives

This article presents a comprehensive review of the current research progress on RSV vaccination in children and pregnant women. The findings of this review offer valuable insights and novel ideas for the future development of RSV vaccines. Despite recent advancements in RSV vaccines, four noteworthy concerns remain: first, the duration of the protective effect of RSV vaccines, which has been reported to last up to one year with vaccines such as MK1654, but requires further investigation to determine their long-term efficacy; second, while successful outcomes have been observed with RSV vaccines for elderly individuals, maternal immunization, and infant monoclonal antibody therapy, children aged 6 months to 5 years still require protection; third, the cooccurrence of RSV and multiple influenza virus epidemic seasons results in infants frequently experiencing multiple viral infections. The efficacy and safety of administering RSV and influenza vaccines concurrently necessitates further substantiation through additional data. Fourth, although maternal-fetal immunity serves as an effective approach for safeguarding newborns, the potential influence of prenatal vaccination on fetal development warrants further scrutiny.

## Author contributions

XG: Data curation, Formal analysis, Investigation, Writing – original draft. EL: Data curation, Formal analysis, Methodology, Validation, Writing – original draft. LF: Data curation, Formal analysis, Investigation, Writing – original draft. WZ: Data curation, Investigation, Methodology, Supervision, Writing – original draft. YY: Writing – review & editing. YD: Funding acquisition, Writing – original draft. XY: Funding acquisition, Writing – review & editing. SX: Validation, Writing – original draft, Writing – review & editing, Funding acquisition.

## References

[1] Linder KA Malani PN. Respiratory syncytial virus. JAMA (2017) 317(1):98. doi: 10.1001/jama.2016.17882 28030703

[2] Ginsburg AS Srikantiah P. Respiratory syncytial virus: promising progress against a leading cause of pneumonia. Lancet Glob Health (2021) 9(12):e1644–5. doi: 10.1016/S2214-109X(21)00455-1 PMC8585487 34774184

[3] Nair H Nokes DJ Gessner BD Dherani M Madhi SA Singleton RJ. Global burden of acute lower respiratory infections due to respiratory syncytial virus in young children: a systematic review and meta-analysis. Lancet (2010) 375(9725):1545–55. doi: 10.1016/S0140-6736(10)60206-1 PMC2864404 20399493

[4] Collins PL Hill MG Cristina J Grosfeld H. Transcription elongation factor of respiratory syncytial virus, a nonsegmented negative-strand RNA virus. Proc Natl Acad Sci USA (1996) 93(1):81–5. doi: 10.1073/pnas.93.1.81 PMC40182 8552680

[5] Kleiner VA Fearns R. RSV M2-1 protein in complex with RNA: old questions are answered and a new one emerges. Structure (2020) 28(9):977–8. doi: 10.1016/j.str.2020.08.007 32877647

[6] Bermingham A Collins PL. The M2-2 protein of human respiratory syncytial virus is a regulatory factor involved in the balance between RNA replication and transcription. Proc Natl Acad Sci USA (1999) 96(20):11259–64. doi: 10.1073/pnas.96.20.11259 PMC18021 10500164

[7] Bitko V Shulyayeva O Mazumder B Musiyenko A Ramaswamy M Look DC. Nonstructural proteins of respiratory syncytial virus suppress premature apoptosis by an NF-kappaB-dependent, interferon-independent mechanism and facilitate virus growth. J Virol (2007) 81(4):1786–95. doi: 10.1128/JVI.01420-06 PMC1797585 17151097

[8] Spann KM Tran KC Collins PL. Effects of nonstructural proteins NS1 and NS2 of human respiratory syncytial virus on interferon regulatory factor 3, NF-kappaB, and proinflammatory cytokines. J Virol (2005) 79(9):5353–62. doi: 10.1128/JVI.79.9.5353-5362.2005 PMC1082743 15827150

[9] Magro M Mas V Chappell K Vázquez M Cano O Luque D. Neutralizing antibodies against the preactive form of respiratory syncytial virus fusion protein offer unique possibilities for clinical intervention. Proc Natl Acad Sci USA (2012) 109(8):3089–94. doi: 10.1073/pnas.1115941109 PMC3286924 22323598

[10] Ngwuta JO Chen M Modjarrad K Joyce MG Kanekiyo M Kumar A. Prefusion F-specific antibodies determine the magnitude of RSV neutralizing activity in human sera. Sci Transl Med (2015) 7(309):309ra162. doi: 10.1126/scitranslmed.aac4241 PMC4672383 26468324

[11] Krarup A Truan D Furmanova-Hollenstein P Bogaert L Bouchier P Bisschop IJM. A highly stable prefusion RSV F vaccine derived from structural analysis of the fusion mechanism. Nat Commun (2015) 6:8143. doi: 10.1038/ncomms9143 PMC4569726 26333350

[12] McLellan JS Chen M Joyce MG Sastry M Stewart-Jones GBE Yang Y. Structure-based design of a fusion glycoprotein vaccine for respiratory syncytial virus. Science (2013) 342(6158):592–8. doi: 10.1126/science.1243283 PMC4461862 24179220

[13] Battles MB McLellan JS. Respiratory syncytial virus entry and how to block it. Nat Rev Microbiol (2019) 17(4):233–45. doi: 10.1038/s41579-019-0149-x PMC7096974 30723301

[14] Mazur NI Terstappen J Baral R Bardají A Beutels P Buchholz UJ. Respiratory syncytial virus prevention within reach: the vaccine and monoclonal antibody landscape. Lancet Infect Dis (2023) 23(1):e2–e21. doi: 10.1016/S1473-3099(22)00291-2 35952703 PMC9896921

[15] O'Brien KL Chandran A Weatherholtz R Jafri HS Griffin MP Bellamy T. Efficacy of motavizumab for the prevention of respiratory syncytial virus disease in healthy Native American infants: a phase 3 randomised double-blind placebo-controlled trial. Lancet Infect Dis (2015) 15(12):1398–408. doi: 10.1016/S1473-3099(15)00247-9 26511956

[16] Fernandez P Trenholme A Abarca K Griffin MP Hultquist M Harris B. A phase 2, randomized, double-blind safety and pharmacokinetic assessment of respiratory syncytial virus (RSV) prophylaxis with motavizumab and palivizumab administered in the same season. BMC Pediatr (2010) 10:38. doi: 10.1186/1471-2431-10-38 20525274 PMC2898783

[17] Griffin MP Khan AA Esser MT Jensen K Takas T Kankam MK. Safety, tolerability, and pharmacokinetics of MEDI8897, the respiratory syncytial virus prefusion F-targeting monoclonal antibody with an extended half-life, in healthy adults. Antimicrob Agents Chemother (2017) 61(3):e01714–16. doi: 10.1128/AAC.01714-16 PMC5328523 27956428

[18] Griffin MP Yuan Y Takas T Domachowske JB Madhi SA Manzoni P. Single-dose nirsevimab for prevention of RSV in preterm infants. N Engl J Med (2020) 383(5):415–25. doi: 10.1056/NEJMoa1913556 32726528

[19] Domachowske J Madhi SA Simões EAF Atanasova V Cabañas F Furuno K. Safety of Nirsevimab for RSV in infants with heart or lung disease or prematurity. N Engl J Med (2022) 386(9):892–4. doi: 10.1056/NEJMc2112186 35235733

[20] Hammitt LL Dagan R Yuan Y Cots MB Bosheva M Madhi SA. Nirsevimab for prevention of RSV in healthy late-preterm and term infants. N Engl J Med (2022) 386(9):837–46. doi: 10.1056/NEJMoa2110275 35235726

[21] Karron RA Luongo C Mateo JS Wanionek K Collins PL Buchholz UJ. Safety and immunogenicity of the respiratory syncytial virus vaccine RSV/DeltaNS2/Delta1313/I1314L in RSV-seronegative children. J Infect Dis (2020) 222(1):82–91. doi: 10.1093/infdis/jiz408 31605113 PMC7199783

[22] Cunningham CK Karron RA Muresan P Kelly MS McFarland EJ Perlowski C. Evaluation of recombinant live-attenuated respiratory syncytial virus (RSV) vaccines RSV/deltaNS2/delta1313/I1314L and RSV/276 in RSV-seronegative children. J Infect Dis (2022) 226(12):2069–78. doi: 10.1093/infdis/jiac253 PMC10205613 35732186

[23] McFarland EJ Karron RA Muresan P Cunningham CK Valentine ME Perlowski C. Live-attenuated respiratory syncytial virus vaccine candidate with deletion of RNA synthesis regulatory protein M2-2 is highly immunogenic in children. J Infect Dis (2018) 217(9):1347–55. doi: 10.1093/infdis/jiy040 PMC5894092 29509911

[24] McFarland EJ Karron RA Muresan P Cunningham CK Perlowski C Libous J. Live-attenuated respiratory syncytial virus vaccine with M2-2 deletion and with small hydrophobic noncoding region is highly immunogenic in children. J Infect Dis (2020) 221(12):2050–9. doi: 10.1093/infdis/jiaa049 PMC7289559 32006006

[25] Buchholz UJ Cunningham CK Muresan P Gnanashanmugam D Sato P Siberry GK. Live respiratory syncytial virus (RSV) vaccine candidate containing stabilized temperature-sensitivity mutations is highly attenuated in RSV-Seronegative infants and children. J Infect Dis (2018) 217(9):1338–46. doi: 10.1093/infdis/jiy066 PMC5894088 29509929

[26] Bebia Z Reyes O Jeanfreau R Kantele A De Leon RG Sánchez MG. Safety and immunogenicity of an investigational respiratory syncytial virus vaccine (RSVPreF3) in mothers and their infants: a phase 2 randomized trial. J Infect Dis (2023) 228(3):299–310. doi: 10.1093/infdis/jiad024 PMC10420396 36722147

[27] Schwarz TF Johnson C Grigat C Apter D Csonka P Lindblad N. Three dose levels of a maternal respiratory syncytial virus vaccine candidate are well tolerated and immunogenic in a randomized trial in nonpregnant women. J Infect Dis (2022) 225(12):2067–76. doi: 10.1093/infdis/jiab317 PMC9200160 34146100

[28] Simoes EAF Center KJ Tita ATN Swanson KA Radley D Houghton J. Prefusion F protein-based respiratory syncytial virus immunization in pregnancy. N Engl J Med (2022) 386(17):1615–26. doi: 10.1056/NEJMoa2106062 35476650

[29] Peterson JT Zareba AM Fitz-Patrick D Essink BJ Scott DA Swanson KA. Safety and immunogenicity of a respiratory syncytial virus prefusion F vaccine when coadministered with a tetanus, diphtheria, and acellular pertussis vaccine. J Infect Dis (2022) 225(12):2077–86. doi: 10.1093/infdis/jiab505 PMC9200146 34637519

[30] Kampmann B Madhi SA Munjal I Simões EAF Pahud BA Llapur C. Bivalent prefusion F vaccine in pregnancy to prevent RSV illness in infants. N Engl J Med (2023) 388(16):1451–64. doi: 10.1056/NEJMoa2216480 37018474

[31] Schwarz TF McPhee RA Launay O Leroux-Roels G Talli J Picciolato M. Immunogenicity and safety of 3 formulations of a respiratory syncytial virus candidate vaccine in nonpregnant women: A phase 2, randomized trial. J Infect Dis (2019) 220(11):1816–25. doi: 10.1093/infdis/jiz395 PMC6898794 31418022

[32] Beran J Lickliter JD Schwarz TF Johnson C Chu L Domachowske JB. Safety and immunogenicity of 3 formulations of an investigational respiratory syncytial virus vaccine in nonpregnant women: results from 2 phase 2 trials. J Infect Dis (2018) 217(10):1616–25. doi: 10.1093/infdis/jiy065 PMC5913599 29401325

[33] Madhi SA Polack FP Piedra PA Munoz FM Trenholme AA Simões EAF. Respiratory syncytial virus vaccination during pregnancy and effects in infants. N Engl J Med (2020) 383(5):426–39. doi: 10.1056/NEJMoa1908380 PMC7299433 32726529

[34] Munoz FM Swamy GK Hickman SP Agrawal S Piedra PA Glenn GM. Safety and immunogenicity of a respiratory syncytial virus fusion (F) protein nanoparticle vaccine in healthy third-trimester pregnant women and their infants. J Infect Dis (2019) 220(11):1802–15. doi: 10.1093/infdis/jiz390 31402384

[35] Cingoz O. Motavizumab. MAbs (2009) 1(5):439–42. doi: 10.4161/mabs.1.5.9496 PMC2759493 20065632

[36] Pantaleo G Correia B Fenwick C Joo VS Perez L. Antibodies to combat viral infections: development strategies and progress. Nat Rev Drug Discovery (2022) 21(9):676–96. doi: 10.1038/s41573-022-00495-3 PMC9207876 35725925

[37] Mousa JJ Kose N Matta P Gilchuk P Crowe JE Jr. A novel pre-fusion conformation-specific neutralizing epitope on the respiratory syncytial virus fusion protein. Nat Microbiol (2017) 2:16271. doi: 10.1038/nmicrobiol.2016.271 28134924 PMC5463187

[38] Su C Zhong Y Zhao G Hou J Zhang S Wang B. RSV pre-fusion F protein enhances the G protein antibody and anti-infectious responses. NPJ Vaccines (2022) 7(1):168. doi: 10.1038/s41541-022-00591-w 36535957 PMC9762623

[39] Kaler J Hussain A Patel K Hernandez T Ray S. Respiratory syncytial virus: A comprehensive review of transmission, pathophysiology, and manifestation. Cureus (2023) 15(3):e36342. doi: 10.7759/cureus.36342 37082497 PMC10111061

[40] Subramanian KN Weisman LE Torunn R Ronald A Sánchez Pablo J Jean S. Safety, tolerance and pharmacokinetics of a humanized monoclonal antibody to respiratory syncytial virus in premature infants and infants with bronchopulmonary dysplasia. MEDI-493 Study Group. Pediatr Infect Dis J (1998) 17(2):110–5. doi: 10.1097/00006454-199802000-00006 9493805

[41] Palivizumab, a humanized respiratory syncytial virus monoclonal antibody, reduces hospitalization from respiratory syncytial virus infection in high-risk infants. The IMpact-RSV Study Group. Pediatrics (1998) 102(3 Pt 1):531–7. 9724660

[42] joint statement with the, F. and C. Newborn, *Palivizumab and respiratory syncytial virus immune globulin intravenous for the prophylaxis of respiratory syncytial virus infection in high risk infants* . Paediatr Child Health (1999) 4(7):474–89. PMC2827759 20212962

[43] Johnson S Oliver C Prince GA Hemming VG Pfarr DS Wang S-C. Development of a humanized monoclonal antibody (MEDI-493) with potent in vitro and in vivo activity against respiratory syncytial virus. J Infect Dis (1997) 176(5):1215–24. doi: 10.1086/514115 9359721

[44] Wu H Pfarr DS Tang Y An L-L Patel NK Watkins JD. Ultra-potent antibodies against respiratory syncytial virus: effects of binding kinetics and binding valence on viral neutralization. J Mol Biol (2005) 350(1):126–44. doi: 10.1016/j.jmb.2005.04.049 15907931

[45] Keam SJ. Nirsevimab: first approval. Drugs (2023) 83(2):181–7. doi: 10.1007/s40265-022-01829-6 36577878

[46] Aliprantis AO Wolford D Caro L Maas BM Ma H Montgomery DL. A phase 1 randomized, double-blind, placebo-controlled trial to assess the safety, tolerability, and pharmacokinetics of a respiratory syncytial virus neutralizing monoclonal antibody MK-1654 in healthy adults. Clin Pharmacol Drug Dev (2021) 10(5):556–66. doi: 10.1002/cpdd.883 33125189

[47] Orito Y Otani N Matsumoto Y Fujimoto K Oshima N Maas BM. A phase I study to evaluate safety, pharmacokinetics, and pharmacodynamics of respiratory syncytial virus neutralizing monoclonal antibody MK-1654 in healthy Japanese adults. Clin Transl Sci (2022) 15(7):1753–63. doi: 10.1111/cts.13290 PMC9283748 35506164

[48] Karron RA Buchholz UJ Collins PL. Live-attenuated respiratory syncytial virus vaccines. Curr Top Microbiol Immunol (2013) 372:259–84. doi: 10.1007/978-3-642-38919-1_13 PMC4794267 24362694

[49] Karron RA Atwell JE McFarland EJ Cunningham CK Muresan P Perlowski C. Live-attenuated vaccines prevent respiratory syncytial virus-associated illness in young children. Am J Respir Crit Care Med (2021) 203(5):594–603. doi: 10.1164/rccm.202005-1660OC 32871092 PMC7924568

[50] Billard MN Bont LJ. Live-attenuated respiratory syncytial virus vaccines: time for the next step. Am J Respir Crit Care Med (2021) 203(5):538–9. doi: 10.1164/rccm.202009-3431ED PMC7924581 32986467

[51] Luongo C Winter CC Collins PL Buchholz UJ. Respiratory syncytial virus modified by deletions of the NS2 gene and amino acid S1313 of the L polymerase protein is a temperature-sensitive, live-attenuated vaccine candidate that is phenotypically stable at physiological temperature. J Virol (2013) 87(4):1985–96. doi: 10.1128/JVI.02769-12 PMC3571493 23236065

[52] McFarland EJ Karron RA Muresan P Cunningham CK Libous J Perlowski C. Live respiratory syncytial virus attenuated by M2-2 deletion and stabilized temperature sensitivity mutation 1030s is a promising vaccine candidate in children. J Infect Dis (2020) 221(4):534–43. doi: 10.1093/infdis/jiz603 PMC6996856 31758177

[53] Karron RA Wright PF Belshe RB Thumar B Casey R Newman F. Identification of a recombinant live attenuated respiratory syncytial virus vaccine candidate that is highly attenuated in infants. J Infect Dis (2005) 191(7):1093–104. doi: 10.1086/427813 15747245

[54] Schickli JH Kaur J Tang RS. Nonclinical phenotypic and genotypic analyses of a Phase 1 pediatric respiratory syncytial virus vaccine candidate MEDI-559 (rA2cp248/404/1030DeltaSH) at permissive and non-permissive temperatures. Virus Res (2012) 169(1):38–47. doi: 10.1016/j.virusres.2012.06.027 22771939

[55] Collignon C Bol V Chalon A Surendran N Morel S van den Berg RA. Innate immune responses to chimpanzee adenovirus vector 155 vaccination in mice and monkeys. Front Immunol (2020) 11:579872. doi: 10.3389/fimmu.2020.579872 33329551 PMC7734297

[56] Diez-Domingo J Sáez-Llorens X Rodriguez-Weber MA Epalza C Chatterjee A Chiu C-H. Safety and immunogenicity of a ChAd155-vectored respiratory syncytial virus (RSV) vaccine in healthy RSV-seropositive children 12-23 months of age. J Infect Dis (2023) 227(11):1293–302. doi: 10.1093/infdis/jiac481 PMC10226655 36484484

[57] Falsey AR Williams K Gymnopoulou E Bart S Ervin J Bastian AR. Efficacy and safety of an ad26.RSV.preF-RSV preF protein vaccine in older adults. N Engl J Med (2023) 388(7):609–20. doi: 10.1056/NEJMoa2207566 36791161

[58] Awar M Mylonakis E. In older adults, an Ad26.RSV.preF-RSV preF protein vaccine reduced RSV-related lower respiratory tract disease. Ann Intern Med (2023). doi: 10.7326/J23-0039 37276605

[59] Stuart ASV Virta M Williams K Seppa I Hartvickson R Greenland M. Phase 1/2a safety and immunogenicity of an adenovirus 26 vector respiratory syncytial virus (RSV) vaccine encoding prefusion F in adults 18-50 years and RSV-seropositive children 12-24 months. J Infect Dis (2022) 227(1):71–82. doi: 10.1093/infdis/jiac407 36259542 PMC9796164

[60] Omer SB. Maternal immunization. N Engl J Med (2017) 376(13):1256–67. doi: 10.1056/NEJMra1509044 28355514

[61] Cohen J. Zika rewrites maternal immunization ethics. Science (2017) 357(6348):241. doi: 10.1126/science.357.6348.241 28729493

[62] Zaman K Roy E Arifeen SE Rahman M Raqib R Wilson E. Effectiveness of maternal influenza immunization in mothers and infants. N Engl J Med (2008) 359(15):1555–64. doi: 10.1056/NEJMoa0708630 18799552

